# Construction and Validation of an Oxaliplatin-Resistant Gene Signature in Colorectal Cancer Patients Who Underwent Chemotherapy

**DOI:** 10.3390/ph15091139

**Published:** 2022-09-13

**Authors:** Yixin Yin, Siqi Li, Xinqiang Liang, Kezhi Li, Mingzhi Xie, Bangli Hu

**Affiliations:** 1 Department of Research, Guangxi Medical University Cancer Hospital, Nanning 530021, China; 2 Department of Chemotherapy, Guangxi Medical University Cancer Hospital, Nanning 530021, China

**Keywords:** colorectal cancer, oxaliplatin, chemoresistance, gene signature

## Abstract

Aberrant expression of genes contributes to the chemoresistance of colorectal cancer (CRC) treatment. This study aimed to identify genes associated with the chemoresistance of oxaliplatin-based chemotherapy in CRC patients and to construct a signature. Oxaliplatin resistance-related genes were screened by analyzing the gene profiles of cell lines and tissue samples that underwent oxaliplatin-based treatment. Oxaliplatin resistance-related genes were used to establish a signature. The association of the signature had clinical significance, so the prognostic value of the signature was analyzed. Independent cohorts and CRC cell lines were used to validate the value of the gene signature and the oxaliplatin-resistant genes. There were 64 oxaliplatin resistance-related genes identified after overlapping the genes from the dataset of oxaliplatin-treated CRC cells and the dataset of patients treated with oxaliplatin-based chemotherapy. A gene signature based on five oxaliplatin resistance-related genes was established. This gene signature effectively predicted the prognosis of CRC patients who underwent chemotherapy. No significant associations were found between the gene mutations and survival of the patients; however, two genes were associated with microsatellite instability status. Two external independent cohorts and CRC cell line experiments validated the prognostic values of the signature and expression of the genes after oxaliplatin treatment. In conclusion, the oxaliplatin resistance-related gene signature involving five genes was a novel biomarker for the prediction of the chemotherapy response and prognosis of CRC patients who underwent oxaliplatin-based chemotherapy.

## 1. Introduction

Colorectal cancer (CRC) is the third most common cancer and the second leading cause of cancer-related death in the world [1]. Chemotherapy remains the major treatment for CRC patients, especially those in the later stages. Currently, oxaliplatin-based chemotherapy regimens, such as FOLFOX or FOLFOXIRI, which consist mainly of fluorouracil, oxaliplatin, and leucovorin, remain the first-line chemotherapy for CRC patients in the later stages [2]. However, most patients develop drug resistance to FOLFOX chemotherapy after several periods of treatment, resulting in treatment failure and an increase in chemotherapy-associated toxicities [3]. Therefore, to facilitate the treatment of CRC patients in the later stages, it is imperative to understand the mechanisms of resistance to oxaliplatin-based chemotherapy.

As one of the major components of the FOLFOX or FOLFOXIRI regimens, oxaliplatin, a third-generation platinum-based anticancer drug, induces the formation of intra-strand guanine–guanine and guanine–adenine DNA links in the cancer cells. However, all of the CRC cells eventually develop resistance to oxaliplatin after long-term administration [4,5]. Several mechanisms underlying oxaliplatin resistance have been reported in previous studies in which some genes were found to be associated with oxaliplatin resistance and some were found to predict the treatment response and prognoses of CRC patients [6]. However, due to the molecular heterogeneity of CRC, the mechanisms underlying oxaliplatin resistance remain to be elucidated, and more oxaliplatin resistance-related genes still need to determined [7]. Therefore, it is important to establish a gene signature based on oxaliplatin resistance-related genes for the treatment and the prognosis of CRC.

Gene mutations and the microsatellite instability (MSI) status have been implicated in many human disorders and are also important sources of heterogeneity in CRCs [8,9]. Our study aimed to facilitate individualized prognosis prediction and better treatment options for CRC patients using oxaliplatin-based chemotherapy. Our objectives were to screen the genes related to oxaliplatin-based chemoresistance and establish a gene signature that predicts the survival of the patients who underwent oxaliplatin-based chemotherapy. We also examined the association of the gene signature with MSI and immunotherapy. In addition, CRC cells lines treated with oxaliplatin were used to validate genes related to oxaliplatin resistance.

## 2. Results

### 2.1. Identification of Oxaliplatin Resistance-Related DEGs in CRC Cells and Patients Who Underwent Oxaliplatin-Based Chemotherapy

**Figure 1:**
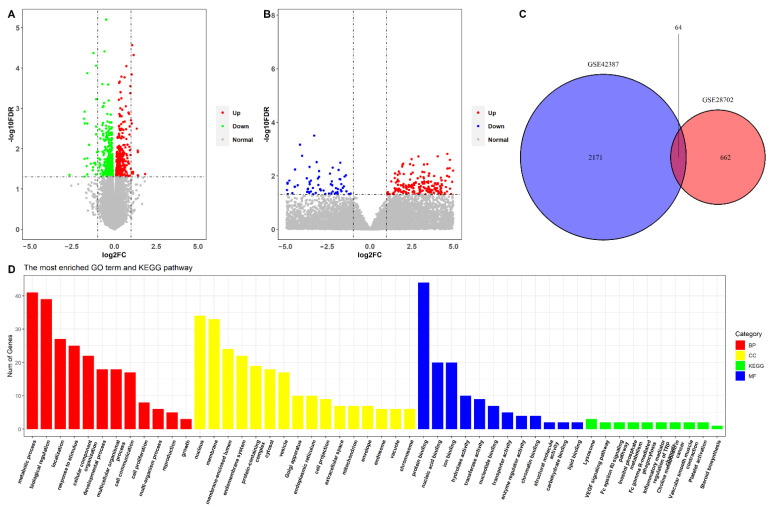
Identification of oxaliplatin resistance-related DEGs. (**A**) Volcano plot of differential expression genes (DEGs) between oxaliplatin-resistant and oxaliplatin-sensitive cells; (**B**) volcano plot of DEGs between non-responder and responder patients who underwent FOLFOX6 chemotherapy; (**C**) Venn plot of the oxaliplatin resistance-related DEGs from cells and clinical samples; (**D**) functional enrichment of oxaliplatin resistance-related DEGs.

The GSE42387 dataset included the data of three CRC cell lines (HCT116, HT-29, and LoVo), and these cells included both the parental cell line and the established oxaliplatin-resistant cell line. We divided the cell lines into parental and oxaliplatin-resistant groups, and differential expression analysis was conducted between the two groups. Based on the screen criteria, a total of 726 differentially expressed genes (DEGs) were identified between the parental and oxaliplatin-resistant groups (Figure 1A). There were 340 upregulated and 386 downregulated genes in the oxaliplatin-resistant group. 

The GSE28702 dataset provided the data of 83 patients with unresectable CRC undergoing FOLFOX therapy, including 41 non-responders and 42 responders. The mean age of the patients was 63.0 ± 10.3 years, with 54 male and 29 female patients. A responder was defined as either a complete response (CR) or a partial response (PR), and a non-responder was defined as a stable disease (SD) or a progressive disease (PD); this definition is according to the Response Evaluation Criteria in Solid Tumors [10]. Assessment of responses to FOLFOX6 therapy was conducted by CT scan after four cycles of therapy. By dividing the patients into responder and non-responder groups, we identified 2235 DEGs in the two groups based on the selection thresholds (Figure 1B), with 514 upregulated and 1721 downregulated genes in the non-responder group. After overlapping the DEGs from the oxaliplatin-treated cells with that of the patients who underwent FOLFOX6 treatment, 64 DEGs that were related to oxaliplatin resistance in both cells and patients were identified (Figure 1C). 

Thereafter, functional enrichment analysis was performed on the 64 oxaliplatin resistance-related DEGs. As shown in Figure 1D, there were 12 significantly enriched BPs, including metabolic processes, biological regulation, and localizations; 16 significantly enriched CCs, including the nucleus and membrane; 12 significantly enriched MFs, including protein binding, nucleic acid binding, and ion binding; and 10 significantly enriched KEGG pathways, including the lysosome, the VEGF signaling pathway, and the Fc epsilon RI signaling pathways. 

### 2.2. Construction of the Gene Signature to Predict the Prognosis of CRC Patients Who Underwent Chemotherapy 

**Figure 2:**
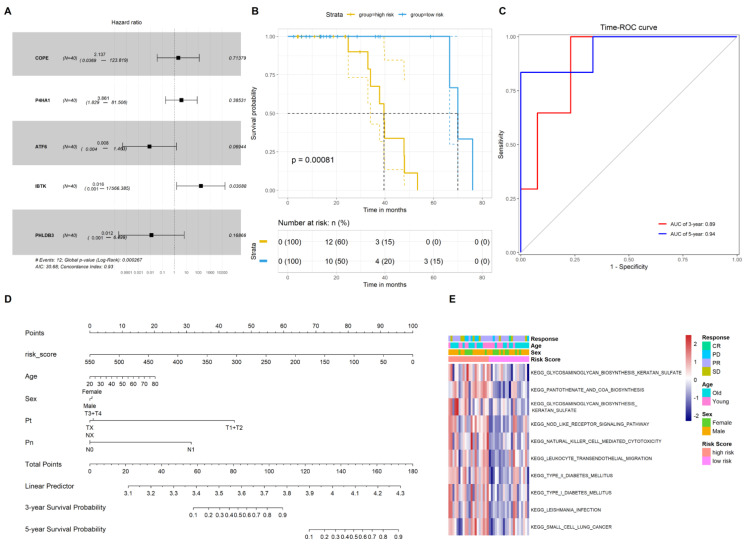
Identification of gene signatures associated with the prognosis of patients who underwent chemotherapy. (**A**) Five oxaliplatin resistance related genes were identified with the prognosis of CRC patients; (**B**) Kaplan–Meier plot revealed the survival time between high- and low-risk scores of gene signatures; (**C**) prognostic values of gene signatures for the 1-, 3-, and 5-year survival of patients; (**D**) nomogram plot of the gene signatures and other clinical features with the prognostic values of patients; (**E**) GSVA algorithm revealed the pathways that gene signatures were involved in.

The samples that were treated with oxaliplatin (FOLFOXIRI and FOLFOX regime) from the GSE72970 dataset were selected, and 40 samples with the survival data were included in the analysis. The Cox regression model was subsequently applied by incorporating the 64 oxaliplatin-resistant DEGs, thus screening the oxaliplatin resistance-related genes that were associated significantly with the survival of CRC patients who underwent chemotherapy. Five genes (*COPE*, *P4HA1*, *ATF6*, *IBTK*, and *PHLDB3*) were significantly associated with poor prognosis (Figure 2A). A prognostic gene signature was then established by calculating the risk scores for the five oxaliplatin resistance-related genes.

Using the median value of the risk scores, 40 CRC patients were stratified into high-and low-risk score groups. Kaplan–Meier analysis indicated that patients in the high-risk score group presented a significantly poorer OS compared with the patients in the low-risk score group (HR = 2.11, log-rank *p* < 0.001, Figure 2B). The time-ROC results revealed that the risk scores had high prognostic values regarding 3- and 5-year survival (Figure 2C). A nomogram demonstrated that the risk score had a better prognostic value than the patients’ age, sex, T stage, and N stage (Figure 2D).

Thereafter, we conducted the GSVA analysis based on the median values of the risk scores. The GSVA was used to explore the molecular pathways and underlying mechanisms related to oxaliplatin resistance in CRC patients. The results showed that genes occurring in the signature in the high-risk score group were mainly related to steroid and primary bile acid biosynthesis as well as to glycosaminoglycan keratin sulfate biosynthetic pathways (Figure 2E).

### 2.3. Mutation and MSI Status of the Five Genes in CRC

**Figure 3:**
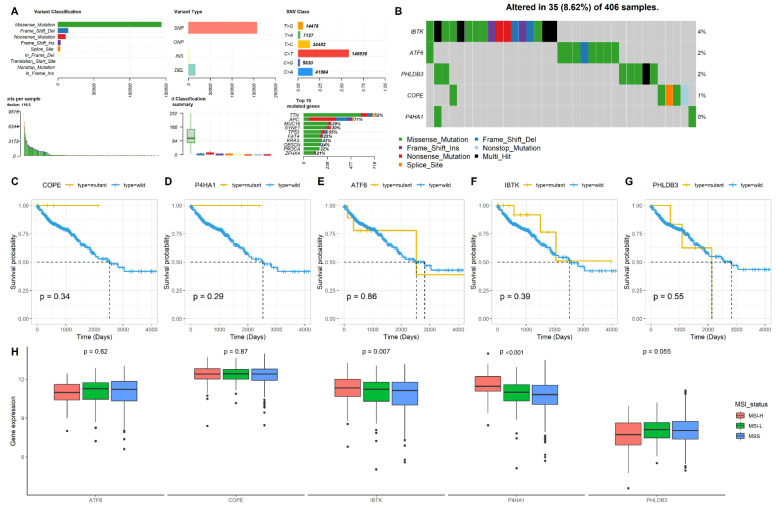
The mutation and MSI status of the five genes in CRC. (**A**) The mutation landscape of genes in CRC; (**B**) the mutation landscape of the five genes in CRC; (**C**–**H**) the association of the five genes’ mutations with the survival of CRC patients; (**H**) Association of the five genes’ expressions with the MSI status of CRC patients.

The gene mutation and the MSI status have been reported to be associated with the development of chemotherapy resistance in CRCs. Therefore, we determined the mutation and MSI patterns of the five genes (*COPE*, *P4HA1*, *ATF6*, *IBTK*, and *PHLDB3*) in CRCs by analyzing the dataset from the TCGA database using the “maftools” package. First, we analyzed the five gene mutation patterns. Figure 3A,B show the mutation landscape of the five genes in CRC, which indicated that the mutations occur less in these five genes. Next, we explored the association of the mutation status of each gene with the survival of CRC patients. Kaplan-Meier analysis revealed that none of the mutant types of any of the five genes showed a significant association with the survival of CRC patients when compared with the wild types (Figure 3C–G). Finally, we determined the association of gene expressions with MSI statuses, including MSI-H, MSI-L, and microsatellite stable (MSS). The results showed that the *P4HA1* and *IBTK* expressions increased in the MSI-H status compared with the MSI-L and MSS statuses (*p* < 0.05), but *COPE*, *ATF6*, and *PHLDB3* failed to show this association (Figure 3H).

### 2.4. Validation of the Gene Signature in Two Independent Datasets

**Figure 4:**
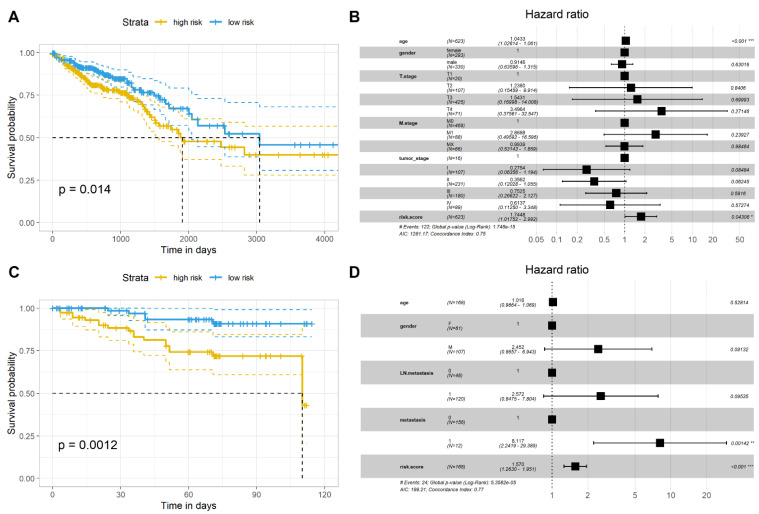
Validation of the gene signature in two independent datasets. (**A**) Kaplan–Meier plot revealed the survival time between high- and low-risk scores of gene signatures in the GSE87211 dataset; (**B**) the forest plot of clinical features and gene signatures in the prediction of the prognosis of CRC patients in the GSE87211 dataset; (**C**) Kaplan–Meier plot revealed the survival time between high- and low-risk scores of gene signatures in the TCGA-COADRAED dataset; (**D**) the forest plot of clinical features and gene signatures in the prediction of the prognosis of CRC patients in the TCGA-COADRAED dataset. * *p* < 0.05, ** *p* < 0.01,*** *p* < 0.001.

To verify the prognostic value of the gene signature in CRC patients, we used two independent datasets, the GSE87211 dataset and the TCGA-COADRAED dataset. In the GSE87211 dataset, we selected only oxaliplatin-treated samples (*n* = 168). Similar to the results of the GSE72970 dataset, the results from the GSE87211 dataset and the TCGA dataset revealed that patients in the high-risk group had poor prognoses compared with those in the low-risk group (Figure 4A,B). The forest graphs further showed that the gene signature had a better prognostic value compared with the TNM and clinical stages of CRC (Figure 4C,D). 

### 2.5. Validation of the Five Oxaliplatin-Resistant Genes in Three CRC Cells

**Figure 5:**
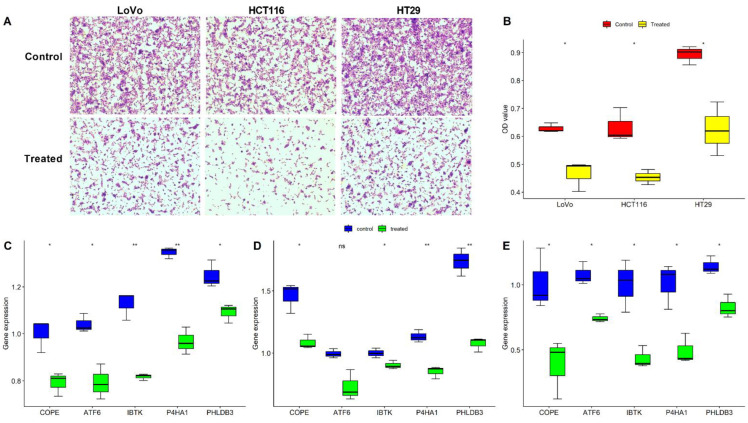
Validation of the five oxaliplatin-resistant genes in HCT116, HT-29, and LoVo CRC cells. (**A**) Cell proliferation morphology after treatment with oxaliplatin for 24 h; (**B**) CCK-8 assay revealing the cell proliferation after treatment with oxaliplatin; comparison of the five oxaliplatin-resistant gene expressions after treatment with oxaliplatin tested by RT-PCR assay in (**C**) HCT116, (**D**) HT-29, and (**E**) LoVo. * *p* < 0.05, ** *p* < 0.01, ns: not significant.

The three CRC cell lines (HCT116, HT-29, and LoVo) were cultured and treated with oxaliplatin for 24 h, and then cell proliferation was examined using the CCK8 assay. Expression of the five genes (*COPE*, *P4HA1*, *ATF6*, *IBTK*, and *PHLDB3*) was tested using the RT-PCR method. Figure 5A,B show that the proliferation of the three CRC cells was inhibited significantly after being treated with oxaliplatin, and the mRNA expression of *COPE, P4HA1, ATF6, IBTK, and PHLDB3* showed a large change in the oxaliplatin-treated group compared with the control groups in all three cell lines (Figure 5C–E). Collectively, these results indicated that the five genes were associated with the effect of oxaliplatin treatment in CRC cells.

## 3. Discussion

Chemoresistance of oxaliplatin-based chemotherapy remains a severe obstacle in the treatment of CRC patients. Although the causes of chemoresistance are complex, aberrant gene expression is considered to be closely related to it [11]; therefore, evaluation of gene expression to identify reliable prognostic biomarkers for oxaliplatin-based chemotherapy is reasonable. In the present study, we firstly screened the genes related to oxaliplatin-based chemotherapy by analyzing datasets from clinical samples and cells, and 64 oxaliplatin-resistant genes were identified. We then used the five oxaliplatin-resistant genes to establish an oxaliplatin-resistant gene signature. This gene signature provided a better prognostic value for the survival of CRC patients who underwent oxaliplatin-based chemotherapy. Furthermore, the results failed to show a relationship with respect to the mutations of the five genes with the survival of CRC patients, although two genes were associated with the MSI status. Finally, we validated the prognostic value of the gene signature using two independent cohorts and verified the expressions of the five genes in three CRC cell lines. These results demonstrate the role of the gene signature and each gene in the resistance of oxaliplatin-based chemotherapy. 

Oxaliplatin is a crucial chemotherapeutic agent in the management of patients suffering mainly from CRC and other tumors. Therefore, it is important to elucidate the molecular mechanisms underlying the resistance phenomena, the main cause of treatment failure, and the progression of CRC. Previous studies have identified several genes that contributed to oxaliplatin resistance, such as *ABCB1/MDR1* [12], *BMAL1* [13], *TYMS*, and *MTHFR* [3]. Since aberrant gene change is associated with oxaliplatin resistance, a gene signature that consists of some of these genes may result in a more reliable predictive value compared with the use of a single gene. Recently, a study using an oxaliplatin resistance-related gene signature (consisting of *CD22*, *CASP1*, *CISH*, and *ALCAM*) to predict the survival of patients with colon cancer found that this gene signature has a better predictive value [6]. However, oxaliplatin resistance is affected by many genes; thus, the potential of genes that participate in oxaliplatin resistance needs to be explored further. However, no study has determined the role of the above five genes in oxaliplatin resistance. Therefore, the role of these five genes in oxaliplatin resistance should be examined in future studies. 

In the present study, we identified five genes with overlapping DEGs between the CRC cells and FOLFOX regime, and these genes were all associated with the survival of CRC patients who underwent oxaliplatin-based chemotherapy. Recently, P4HA1 was reported to regulate human CRC cells through the HIF1α-mediated Wnt signaling pathway [14]. Targeting of P4HA1 with diethyl-pythiDC could be an effective therapeutic strategy for aggressive CRCs [15]. Recently, there was study reporting that the P4HA1 expression was increased in clear cell renal cell carcinoma compared to adjacent normal tissues [16], and P4HA1 was substantially overexpressed in 26 of 33 cancers types, including liver cancer, pancreatic cancer, and stomach cancer; P4HA1 overexpression was associated with poor survival in these patients [17]. Furthermore, ATF6 has been reported to facilitate the development of CRC, and ATF6 activation was shown to result in CRC cell proliferation and a reduction in the expression of markers of intestinal epithelial stemness in CRC [18]. PHLDB3 was also shown to promote colon cancer cell growth by inactivating p53 in a negative feedback fashion [19]. Although no study explored the role of IBTK in CRC, one study has shown that IBTK contributed to the B-cell lymphomagenesis in Emu-myc transgenic mice that conferred a resistance to apoptosis [20]. With regard to COPE, which is also termed COPI coat complex subunit epsilon, a study showed that the silencing of COPB2 inhibits CRC cell proliferation and induces apoptosis via the JNK/c-Jun signaling pathway [21].

MSI has been shown to be associated with the chemotherapy response and prognosis of CRC patients, and adding oxaliplatin to fluoropyrimidine improves the survival of colon cancer patients with MSI stage III [22]. One study reported that the effect of fluoropyrimidine/oxaliplatin first-line chemotherapy was not different between MSI-H and MSI-L/MSS tumors, and MSI-H tumors tended to have better prognosis [23]. The present study showed that only *IBTK* and *P4HA1* expressions were associated with the MSI status, suggesting that these two genes might contribute to the effect of oxaliplatin-based chemotherapy in CRC patients with different MSI statuses.

Compared to previous studies, the present study selected the genes from the overlapped oxaliplatin-treated cells and the FOLFOX chemotherapy, which were specifically associated with oxaliplatin resistance. Furthermore, the prognostic value of the gene signature was validated using two independent cohorts, indicating the robustness of the results. Finally, we validated the expression of each gene in the oxaliplatin-treated cells. These results highlighted the clinical significance of the gene signature and the role of each gene in oxaliplatin-based chemotherapy. 

However, there were several limitations in this study. First, the GSE28702 dataset that was used to screen DEGs lacked data from patients who initially responded and then progressed, as well as tissue from both time points; accordingly, we could not identify the progressive selection of resistance genes under continued FOLFOX treatment. Second, considering that the FOLFOX regimen consists of oxaliplatin, leucovorin, and fluorouracil and that oxaliplatin is rarely to never administered alone, it is difficult to definitively determine that these treatments specifically resulted in oxaliplatin resistance; they may lead to overall chemoresistance owing to increased drug elimination, cellular proliferation, or heightened resistant to apoptosis rather than an oxaliplatin-specific effect, per se. Therefore, we recommend that the clinical significance of the gene signature be further validated using a larger number of clinical samples, with and without responses to oxaliplatin-based chemotherapy and that the biological function of each gene should be further determined using in vivo and in vitro studies. In addition, we did not develop oxaliplatin-resistant cells to determine the expressions of the five genes; therefore, using oxaliplatin-resistant cells to verify the change in gene expressions is necessary in future studies.

## 4. Materials and Methods

### 4.1. Data Acquisition and Processing

The GSE72970 dataset (124 samples) [24] was downloaded from the GEO database (https://www.ncbi.nlm.nih.gov/gds) (access date: 22 January 2022), which provided the response statuses and survival data of CRC patients who underwent oxaliplatin-based chemotherapy. The chemotherapy regime of the patients included FOLFIRI; FOLFIRI combined with BEVACIZUMAB or ERBITUX; FOLFOXIRI; FOLFOXIRI combined with BEVACIZUMAB; FOLFOX; FOLFOX combined with BEVACIZUMAB; or XELIRI combined with BEVACIZUMAB. We only selected samples (*n* = 40) from patients treated with oxaliplatin (FOLFOXIRI and FOLFOX regime). The expression profile datasets of oxaliplatin-treated CRC cell lines (HCT116, HT-29, and LoVo) were downloaded from the GSE42387 dataset (27 samples) [25]. The data of CRC patients who underwent FOLFOX6 chemotherapy were obtained from the GSE28702 datasets (83 samples) [26]. The GSE87211 dataset (363 samples, including 9 samples of 5-FU + oxaliplatin + cetuximab + RT and 159 samples of 5-FU + oxaliplatin + RT) [27] was downloaded to verify prognosis of the gene signature. Gene expression, mutations, MSI data, and corresponding clinical and follow-up data of the COADREAD patients (367 samples) were downloaded from the UCSC Xena database. Raw data were preprocessed using background adjustments, quantile normalizations, and log2 transformations, as described previously [28]. Differentially expressed genes in the datasets were determined using the “limma” package for the GEO dataset and the “edgeR” package for the TCGA dataset, with *p* < 0.05 as the selection threshold.

### 4.2. Gene Ontology Analysis and Gene Set Variation Analysis (GSVA)

The functions of the oxaliplatin resistance-related genes were analyzed using Gene Ontology (GO) analyses, including biological processes (BPs), molecular functions (MFs), and cellular components (CCs), and the Kyoto Encyclopedia of Genes and Genomes (KEGG) analysis using the WebGestalt online tool [29], with an FDR value < 0.05 considered as statistically significant enrichment. The GSVA was used to determine the most significantly enriched molecular pathways between high- and low-risk groups of gene signatures [30]. 

### 4.3. Construction of an Oxaliplatin Resistance-Related Gene-Based Prognostic Signature

The oxaliplatin resistance-related gene-based prognostic signature was established and characterized using previously reported risk scores [31]. Briefly, the regression coefficient (β) that was derived from the multivariate Cox regression analyses was multiplied by the expression of the corresponding gene to generate the risk score according to the following formula: risk score = (β mRNA1 × expression of mRNA1) + (β mRNA2 × expression of mRNA2) +…+ (β mRNAn × expression of mRNAn). Thereafter, the patients were divided into high- and low-risk signature groups based on the median value of the risk scores. Next, the association of the gene signature with patients’ survival and clinical features was determined. These results were further validated in the TCGA-COADREAD and GSE87211 datasets.

### 4.4. Cell Culture and Real-Time Polymerase Chain Reaction (RT-PCR) Analysis

Three CRC cell lines (HCT116, LoVo, and HT-29) were obtained from the Shanghai Cell Bank of Chinese Academy of Sciences (Shanghai, China). These cell lines were negative for mycoplasma infection and cultured in DMEM with GlutaMAX (Gibco, Brooklyn, NY, USA) supplemented with 10% FBS and 1% streptomycin/penicillin. Oxaliplatin was purchased from Selleck Chemical (Selleck, S1224, TX, USA) and dissolved in dimethyl sulfoxide (DMSO, MP Biomedicals, Irvine, CA, USA) at a concentration of 35 μmol/L for 24 h. The CRC cells were seeded in 96-well plates at a density of 2 × 10^3^ cells per well. A Cell Counting Kit-8 (CCK8) solution (Biosharp, BS350B, Hefei, Anhui, China) was added to each well prior to incubation at 37 °C for 2 h. Total RNA from CRC tissues and cell lines was isolated using TRIzol reagent (Invitrogen; Thermo Fisher Scientific, Inc., Waltham, MA, USA). First-strand cDNA was synthesized using reverse transcription from 1 µg of RNA using the PrimeScript RT reagent Kit with gDNA Eraser (Takara, Dalian, China). RT-PCR was performed using the SYBR^®^ Premix Ex Taq kit (Takara, Dalian, China) following a standard protocol based on the manufacturer’s instructions. *GAPDH* was used as an internal control. Gene expression was quantified using the 2^−ΔΔCT^ method [32]. Gene primers used for the RT-PCR are listed in Supplementary Table S1.

### 4.5. Statistical Analysis

All statistical analyses were performed using R software (Version: 3.6.5). An independent Student’s *t*-test for continuous data was used for comparisons between the two groups. The Mann–Whitney U-test was used to compare non-normally distributed variables. Progression-free survival (PFS) and overall survival (OS) between the two groups were compared using Kaplan–Meier analysis and the log-rank test. Univariate and multivariate analyses were conducted using the Cox proportional regression model. A two-tailed *p*-value < 0.05 was considered to be statistically significant. 

## 5. Conclusions

This study screened genes related to oxaliplatin resistance, established an oxaliplatin resistance signature based on five genes, and validated its clinical significance and prognostic value in CRC patients who underwent oxaliplatin-based chemotherapy. Our study provided insights into oxaliplatin-resistant gene expression in CRCs; these findings can help identify CRC patients who are suitable for oxaliplatin-based chemotherapy. 

## Data Availability

Data is contained within the article and supplementary material.

## Supplementary Materials

The following supporting information can be downloaded at: https://www.mdpi.com/article/10.3390/ph15091139/s1, Table S1: Primer Sequence of genes.
