# Supplementary material for: Construction and Validation of an Oxaliplatin-Resistant Gene Signature in Colorectal Cancer Patients Who Underwent Chemotherapy
Source: Pharmaceuticals (Basel). 2022 Sep 13;15(9):1139. doi: 10.3390/ph15091139 (PMC9503614; doi:10.3390/ph15091139)
Supplement: Supplementary file 1 [file pharmaceuticals-15-01139-s001.zip › pharmaceuticals-1847493-supplementary.pdf]

Supplement files

Table S1 Primer Sequence of genes

Genes		Primer Sequence
COPE	FORWARD	5’- AGTTCGGTGTGGTCCTGGATGAG-3’
	REVERSE	5’-GTGGGCGAGGTAGTCAGCAAAC-3’
ATF6	FORWARD	5’-TGATGCCTTGGGAGTCAGACATTTG-3’
	REVERSE	5’-CGAGGAGACGAGACTGAATAACTTGAG-3’
IBTK	FORWARD	5’-TATTCCACCGCCTTCCAGTTGTAATG-3’
	REVERSE	5’-CAACGCCAATGATTGTCCTTCCTTTC-3’
PHLDB3	FORWARD	5’-TCAAGACCTGGAGGAAGCGATGG-3’
	REVERSE	5’-GCCTGGAAGTAGATGACACCTTTGAG-3’
P4HA1	FORWARD	5’-CTAACGGCTGAGGACTGCTTTGAG-3’
	REVERSE	5’-CTCGCCTTCATCCAGTTGCCTTAG-3’
GAPDH	FORWARD	5’-GGA CCT CAT GGC CTA CAT GG-3’
	REVERSE	5’-TAG GGC CTC TCT TGC TCA GT-3’
